# Accelerating Translational Research by Clinically Driven Development of an Informatics Platform–A Case Study

**DOI:** 10.1371/journal.pone.0104382

**Published:** 2014-09-09

**Authors:** Imad Abugessaisa, Saedis Saevarsdottir, Giorgos Tsipras, Staffan Lindblad, Charlotta Sandin, Pernilla Nikamo, Mona Ståhle, Vivianne Malmström, Lars Klareskog, Jesper Tegnér

**Affiliations:** 1 Unit of Computational Medicine, Department of Medicine, Center for Molecular Medicine, Karolinska Institutet, Stockholm, Sweden; 2 Rheumatology Unit, Department of Medicine, Center for Molecular Medicine, Karolinska Institutet, Stockholm, Sweden; 3 Medical Management Center, Department of Learning, Informatics, Management and Ethics, Karolinska Institutet, Stockholm, Sweden; 4 Dermatology and Venereology Unit, Department of Medicine, Karolinska Institutet, Karolinska University Hospital, Stockholm, Sweden; University of Groningen, University Medical Center Groningen, Netherlands

## Abstract

Translational medicine is becoming increasingly dependent upon data generated from health care, clinical research, and molecular investigations. This increasing rate of production and diversity in data has brought about several challenges, including the need to integrate fragmented databases, enable secondary use of patient clinical data from health care in clinical research, and to create information systems that clinicians and biomedical researchers can readily use. Our case study effectively integrates requirements from the clinical and biomedical researcher perspectives in a translational medicine setting. Our three principal achievements are (a) a design of a user-friendly web-based system for management and integration of clinical and molecular databases, while adhering to proper de-identification and security measures; (b) providing a real-world test of the system functionalities using clinical cohorts; and (c) system integration with a clinical decision support system to demonstrate system interoperability. We engaged two active clinical cohorts, 747 psoriasis patients and 2001 rheumatoid arthritis patients, to demonstrate efficient query possibilities across the data sources, enable cohort stratification, extract variation in antibody patterns, study biomarker predictors of treatment response in RA patients, and to explore metabolic profiles of psoriasis patients. Finally, we demonstrated system interoperability by enabling integration with an established clinical decision support system in health care. To assure the usefulness and usability of the system, we followed two approaches. First, we created a graphical user interface supporting all user interactions. Secondly we carried out a system performance evaluation study where we measured the average response time in seconds for active users, http errors, and kilobits per second received and sent. The maximum response time was found to be 0.12 seconds; no server or client errors of any kind were detected. In conclusion, the system can readily be used by clinicians and biomedical researchers in a translational medicine setting.

## Introduction

**Figure 1:**
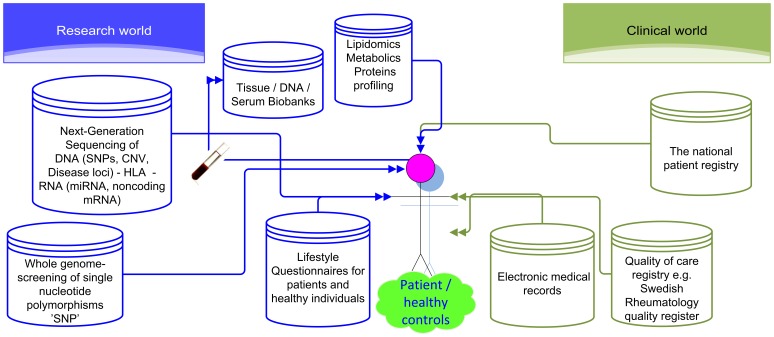
Schematic illustration of different types of database sources that need to be created for the analysis of cases (patients) versus healthy individuals (controls). Since these data are as a rule stored in different domains (clinical and research), a single case-control study needs to collect the data several times during a study, and this cycle must be repeated whenever a new case-control study is initiated.

Translational medicine, aimed at understanding etiology, molecular pathogenesis, clinical features, and prevention and treatment of diseases, depends on quantitative and high-quality data from patients during different stages of disease [1]. To this end, large amounts of clinical data are as a rule captured in electronic medical records (EMR), but increasingly also occasionally in dedicated registries on patients with specific diagnoses, thus capturing information on clinical characteristics of disease, laboratory data, response to therapies, and comorbidities. The success of translational medicine also relies on efficient utilization of data generated from emerging genomics technologies. Hence, to collect and manage large volumes of heterogeneous data has been recognized as a major enabler of translational informatics research [2]. However, unfortunately, these two pillars of translational medicine, clinical records and molecular data, along with their different parts, generally reside in disconnected informatics systems (figure 1). There is therefore an urgent need to reduce these barriers to accessing, sharing, reusing, and analyzing these different sources of data. A development mitigating this gap, thus enabling these data to be searchable across current data silos, would clearly spearhead the development and application of systems [3] and network-based [4], [5] approaches supporting predictive precision medicine, as currently advocated by both the medical [6] and computational research communities [7].

These challenges and opportunities for systems-based translational research have been duly recognized recently. Several parallel efforts have consequently been undertaken to address this unmet need. Open-source initiatives include the i2b2 suite [8], a scalable software platform facilitating repurposing of clinical data into the research setting. This platform has been used to build a system for monitoring clinical trials by combining i2b2 with GenePattern, a suite of bioinformatics tools from Broad Institute [9]. This development has been orchestrated by the pharmaceutical company Johnson and Johnson and the Innovative Medicines Initiative (IMI) eTRIKS project (http://www.imi.europa.eu/content/etriks). However, the resulting system, referred to as tranSMART [10], requires professional software engineering support for the curation and import of data and applications, thus constituting a significant barrier for clinicians. To meet the requirements of clinicians, Stanford Medical Center has developed the STRIDE system [11], to support ongoing clinical research at Stanford University. For a review of these efforts including their pros and cons, and challenges in a broader context, see [12]. In contrast to these open-source or local efforts, a commercial vendor may first of all provide better support and usable graphical user interface (GUI) for clinicians and translational researchers, and secondly provide a secure platform capable of dealing with sensitive patient data in conjunction with molecular data. This possibility has recently been examined; one of the leading commercial platforms on the market, the Oracle Health Sciences Clinical Development Center suite developed by Oracle (http://www.oracle.com/us/corporate/press/350422), was evaluated in a translational research environment [13]. The study concluded that although the system performed well from a technical and usability point of view, there were still pending issues for a hospital or research group. In view of both open-source and commercial efforts, there is therefore ample room for new developments and further investigations of the real needs of clinical users. To this end, our requirements at Karolinska University Hospital, Karolinska Institutet and Stockholm County Council have guided and motivated us to initiate a research and development project aimed at building an informatics system for integrating healthcare and clinical research databases, hence bridging the clinical and research domains as illustrated in figure 1.

Here we show that our study design provides us with a firsthand view of real translational needs from clinical and biomedical users. Our case study describes and evaluates the first version of this system, T-MedFusion, which not only can integrate different kinds of data, thereby enabling translational research, but can also accelerate the research process by providing specific and detailed examples in the area of chronic inflammatory diseases such as psoriasis and rheumatoid arthritis (RA).

## Materials and Methods

The RA study obtained approval from the ethical board at Karolinska Institute, Stockholm, Sweden.

Detailed and technical description of the assembly of methods and open-source software engineering tools used to develop T-MedFusion will be reported in a complementary manuscript. In brief, the T-MedFusion currently contains datasets from different ongoing clinical research studies at Karolinska University Hospital, such as the study of Venous thromboembolism, Non-Small Cell Lung Cancer and chronic inflammatory diseases.

### Understanding translational researchers' data needs

In this section, we will illustrate our approach to acquire and collect end-user's needs and requirements mainly in two therapeutic areas; Psoriasis and Rheumatoid arthritis (RA).

We started by identifying the requirements and types of database sources, the data structure, type of queries and interfaces for clinical researchers, as well as ethical and organization rules and regulations. This initial step helped to define data sources, the storage method, use cases, and experimental platforms used to generate laboratory data. The identification of these components allowed us to create the required architecture for T-MedFusion. After defining the architecture layers of T-MedFusion, we used a prototype as a complementary method to simulate the behavior of the system, enabling end users to refine their ideas and requirements about the system. We developed multiple versions (prototypes) and asked the researchers to use the system to try different types of data extraction, retrieval, patient stratification, and so forth.

The users of T-MedFusion are biomedical and clinical researchers at the Center for Molecular Medicine (CMM), a translational medicine research environment hosting researchers and clinicians affiliated with different departments at the Karolinska Institute working with patient cohorts obtained from the clinical practice under the ruling of the Karolinska University Hospital and Stockholm County Council. To develop and test our method, we selected research groups conducting investigations targeting chronic inflammation diseases with an active cohort of patients at the clinic.

### Clinical and metabolic profiles for psoriasis cases and controls

Psoriasis is a common disease affecting 2–3% of the human population including both men and women [14]. Psoriasis is said to be chronic, but the natural disease course is actually not known. Psoriatic arthritis (PsA) affects one-third of patients over time [15]. PsA is a clinical diagnosis based on clinical findings; at present there are no biomarkers available. Ample epidemiologic evidence supports a link between severe psoriasis and cardiovascular events [16], [17]. Pathogenic mechanisms are incompletely known. Epidemiology also shows that psoriasis associates positively with metabolic dysfunction including obesity and vice versa, i.e., obesity increases the risk of developing psoriasis. However, putative mechanistic links between psoriasis and obesity are unclear. Hence, there is clear interest to perform future investigations across these diseases, thus defining the need for an integrative informatics platform supporting such queries across diseases.

The genetic contribution to psoriasis is overwhelming, and HLA-C is the main associated gene [18]. How HLA-C contributes to disease is still unknown, but it may involve altered presentation of a presumed autoantigen. Several recent genome-wide association studies have identified additional associated genes, the majority of which are involved in the immune system. These studies include unselected patient materials and reveal genes common to major subtypes, whereas genes associated with specific phenotypes may be missed. A striking example is the recent identification of the gene for pustular psoriasis, a rare and severe phenotype, where two independent groups reported mutations in the interleukin-36-receptor antagonist leading to deregulation of inflammation [19], [20]. Psoriasis is immune-mediated and T cells play a prominent role. Depletion of T cells or interference with their activation are effective therapeutic strategies [21]. The hypothesis is that T cells under the influence of dendritic cells drive the disease. Novel treatments targeting such pathways provide powerful tools to understand psoriasis.

To address and investigate the above research questions, a data integration environment is required. The EMR (electronic medical record) of psoriasis patients and the clinical lab results of the cohort of 747 psoriasis patients stored at the dermatology clinic were used. All cases were included within 12 months of onset of skin disease and examined by the dermatologist in the group. All patients with joint irritations were examined by a specialist rheumatologist to ascertain the diagnosis of PsA. The metabolic profile, blood lipids, fasting blood sugar, and body mass index (BMI) of the cohort and the matched controls are stored in different systems at the clinic and CMM. The metabolic profile for each individual in the cohort consists of data collected at onset of disease (during 2000–2004) and 10 years after onset. The latter part of the study is ongoing; to date 225 individuals have been included for follow-up investigation.

### Rheumatoid arthritis (RA)

RA is a chronic inflammatory joint disease. In addition to the synovial joints, RA may affect other organs. RA has a significant effect on the patient's life and employment. In Sweden, the incidence of RA is 0.5–1%; the cumulative prevalence was 0.77% [22].

T-MedFusion enables immunological studies of RA patients, aiming at increasing understanding of disease initiation and perpetuation. To perform such studies, researchers need to take into account and access both the genetic and serological profiles of the included patient material. The patient cohorts diagnosed with RA (defined by ACR 1987 or later ACR/Eular 2010) present three profiles: human leukocyte antigen-DR (HLA-DR) genotyping, genotype of 65 SNPs all predisposing for RA [23] (table 1), and detection of anti citrullinated peptide antibodies (ACPA:s) using the anti-CCP assays or antibodies IgG antibodies against citrullinated alpha-enclose peptide-1 (CEP-1) and citrullinated type-II collagen (citC1I) IgG antibodies against citrullinated vimentin [24], [25].

**Table 1:** List of selected SNPs with their allele frequencies.

		Allele frequencies		
Rs number	Locus	Major	Minor	Risk
6314	HTR2A	C	T	C
1328674	HTR2A	C	T	T
548234	PRDM1	T	C	C
4781003	CIITA	C	T	T
4535211	PLCL2	G	A	A
10431908	CIITA	A	G	G
544167	C2	G	T	G
12746613	FCGR2A	C	T	T
4810485	CD40	G	T	G
10498441	NID2	A	G	A
10499194	OLIG3,TNFAIP3	C	T	C
2064476	HLA-DPB2	A	G	A
706778	IL2RA	C	T	T
2736340	BLK	A	G	G
26232	C5orf30	C	T	C
540386	TRAF6	C	T	C
231707	C4orf8	G	A	A
10402677	CEACAM1	G	A	A
42041	CDK6	C	G	G
2024301	CLEC4A;POU5F1P3	A	T	T
3807306	IRF5	A	C	A
10488631	IRF5;TNPO3	T	C	C
3761847	TRAF1/C5	A	G	G
7026551	C5	A	C	C
11586238	CD2,CD58	C	G	G
231735	CTLA4	G	T	G
13017599	REL	A	G	G
394581	TAGAP	T	C	T
2263484	C21orf74	A	C	C
6682654	CD244			
6859219	ANKRD55	C	A	C
13031237	REL	A	C	C
934734	SPRED2	A	G	G
11676922	AFF3	A	T	T
3087243	CTLA4	G	A	G
1678542	KIF5A	C	G	C
951500	CCL21	A	G	A
892188	GLP-1;FDX1L;ICAM5	C	T	T
1133104	CLEC4A;POU5F1P3	G	T	T
1980422	CD28	T	C	C
1859341	CEACAM8	A	G	G
3087456	CIITA	A	G	G
2271077	GALNTL2	A	G	A
2377422	CLEC4A;POU5F1P3	C	T	T
2476601	PTPN22	C	T	T
2812378	CCL21;C9orf144B	A	G	G
2240340	PADI4	C	T	T
6416647	CIITA	T	C	C
3890745	MMEL1	T	C	T
4272626	NHLH2	C	T	T
10258735	RPA3	A	G	G
3093023	CCR6	G	A	A
3218253	IL2RB	G	A	A
6822844	IL2,IL21	G	T	G
7234029	PTPN2	A	G	G
6457620	HLA-DRA	G	C	G
6920220	OLIG3,TNFAIP3	G	A	A
10413014	CEACAM8	A	G	G
7574865	STAT4	G	T	T
10468473	MAP2K4	G	A	A
10410147	CEACAM8	G	A	A
10919563	PTPRC	G	A	G
4750316	DKFZp667F0711/PRKCQ	G	C	G
2523451	MICA	A	G	G
6457617	HLA-DQ	C	T	C

### Data sources and schemas in T-MedFusion

We started with the identification of data elements in the domain of knowledge (psoriasis and RA), identifying all entities (objects) composing the domain and the relationship among them. We used formal methods to define and model database sources. We used concept mapping [26] as a technique to define concepts and their attributes. Our starting point was therefore to identify and understand the data structure, regulations, and nature of each data source.

### Patient clinical data

For each of the above diseases, the recruited patients have their clinical record stored within the hospital. A previously established system for exchange of structured data between the clinical records and a specific national registry for RA patients (named Swedish Rheumatology Quality of Care Registry (SRQ) was used for transfer of data from the clinical records to the Quality of Care Registry. However, a substantial part of the structured clinical data are also entered directly into the quality of care registry by the respective physicians (for information on the Swedish Rheumatology Quality of Care registry for RA; see [13], [27]. In the present study, the clinical data from RA patients come from patients from Karolinska University Hospital registered in the Swedish Rheumatology Quality Registry. Researchers need to apply specifically for each study to the Register Steering Committee, after ethical approval of the study, to receive coded data from the register in order to perform research using the registry data.

**Figure 2:**
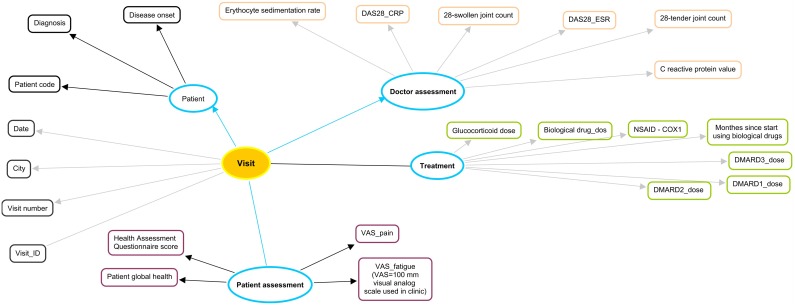
Basic data elements and associated attributes per patient visit to the clinic, as in the Swedish Rheumatology Quality Registry.

In T-MedFusion we modeled and implemented attributes describing the disease duration (temporal), disease activity, and medication for RA patient (figure 2).

**Figure 3:**
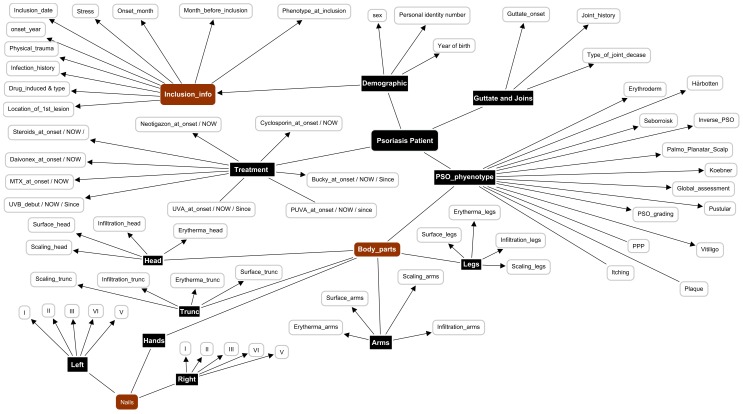
Clinical and metabolic profile of a psoriasis patient.

For the psoriasis patients, clinical phenotypes for the patients were collected at two time points, the first in 2002 and the 10-year follow-up starting in 2012. The basic clinical phenotype includes the development of the disease (healed, guttate, or plaque), psoriasis area severity index (PASI), BMI, low-density lipoprotein (LDL) levels, high-density lipoprotein (HDL) levels, triglyceride (TG) levels, HDL/LDL ratio, general skin examination, nails assessment, treatment, and so forth) (figure 3).

### Biobank and cell register

The biobank and cell registry stores and manages biological specimens from patients and from relevant individuals in the population. Biological specimens are available mainly in the context of specific research into one or several diseases (RA and psoriasis). The biobank contains DNA, RNA, serum, plasma, information on different preparation methods and information on sample volume and of specific information on where the specimen is stored viable cells that are captured and stored from some patients, and the information on these cells is stored in the cell registry database, which contains quantitative information about the number of cells available and other attributes.

### Genotype variants, serology, and autoantibody

The genotype variants and serology data source contains information derived from the biobanked specimens, and these databases contain extensive information concerning the following: genotypes, both HLA-type and extensive SNP data; serology, covering titers of different RA-related autoantibodies (both IgG and IgA), such as CCP (Cyclic Citrullinated Peptide); and fine specificities [24], [25]. For each patient a different measure from the synovial fluid and serum was stored. In addition, reference SNPs are stored and categorized according to gene risk level (Risk, major, minor). See table 1 for the list of selected SNPs with their allele frequencies.

### System usability and interoperability

Through our research and implementation of T-MedFusion we used the iterative process development method, in which we allowed biomedical and clinical researchers to join in the process by testing the user interface and providing us with feedback for a new version.

To assure the usefulness of the system, we decided to consider and explore two complementary approaches. The first approach is to make all user interaction based on a graphical user interface (GUI). The minimum requirements of the interface were to allow clinical and biomedical researchers to perform simple and complex queries on the fused databases. A simple query operates on a single source database, while a complex query retrieves data from fused database sources. In both cases, logical operations are supported. Such operations allow filtering the results according to specific expressions. The user interface enables the retrieval of a single record or group of records for a particular patient or sample. Also, it allows exporting output files into different file formats. The database is aggregated to the patient/sample level. The GUI for SNP data stored in T-MedFusion is illustrated in figure S1 serves as an example of the GUI layout in T-MedFusion.

The second approach is to perform a system performance evaluation study. Here we measure user satisfaction and acceptance of the system behavior based on the result of the performance evaluation. Through the performance evaluation and testing we obtained a quantitative measure to prove the effectiveness of the integration process and identify bottlenecks to overcome.

At the system level, we conducted a performance test to monitor the system's behavior under the following conditions:

- Increase the number of concurrent virtual users and monitor the reaction of the web service to the increased load.
- Operate the test for 120 hours (five days).
- Increase the number of virtual users from 1 to 20.
- Apply matrix and performance results.
- Response time: The elapsed time between the end of any inquiry or demand on a computer system and the beginning of a response (user query or sample selection).
- Average response time (during 120 hours). Maximum response time.
- Percentage of errors (during 120 hours), to identify any type of errors during the integration process.
- Test scenario: The investigator wants to select cells based on HLA type with preconditions of a cell count greater than 50,000,000 and SNP rs2064476 = AG and GG. This scenario involved integration of data from multiple sources available for the integration system. We obtained very good response time per virtual user, and we benchmarked the results with other systems.

**Figure 4:**
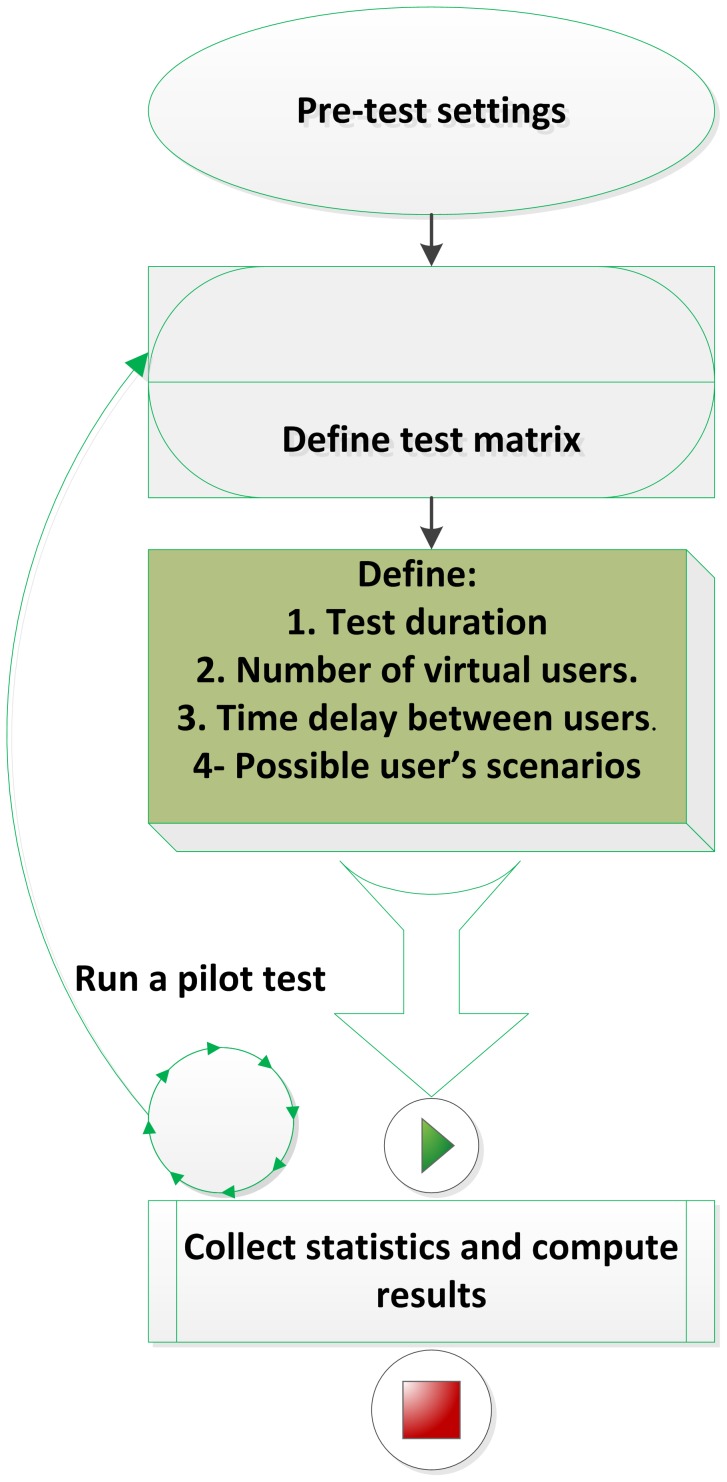
Flowchart depicting the performance evaluation method.

The workflow and the implementation of the performance evaluation methods are illustrated in figure 4. The test was performed on a server running Redhat 5.5 Linux; the server configuration is Windows Intel 1.8 GHZ and 8 GB RAM. The server was running other applications during the test.

T-MedFusion supports interoperability with other systems as well. Through a friendly interface, the user can export the result of the query to many different formats. Importantly, we support integration via web services and provide the database in XML format and schema.

We implemented a web service (WS) in simple object access protocol (SOAP). The advantage of the WS implementation is that it allowed us to integrate T-MedFusion with the clinical decision support system (CDSS), as further elaborated in the Results section. Frequently in research and clinical environments we encounter different kinds of system for different functions. Such systems are developed by different vendors, each as a rule relying upon a different technology platform. A powerful best approach in integrating different software systems is to release WS based on well-defined service contracts in XML format.

## Results

As indicated within the introduction section, T-MedFusion has demonstrated the usefulness and capabilities to handle data from health care, clinical research and molecular data in different therapeutic areas and clinical specialties (Cardiovascular. Oncology, Dermatology and Rheumatology) within Karolinska institute and Karolinska University Hospital.

Here we first we explain the de-identification and security procedures implemented to protect patient and research data. These security requirements were the result of a dialogue between translational researchers and principal biomedical investigators, together with computational researchers. Secondly we report the results from a real-use case study of the system from investigators working on either psoriasis or rheumatoid arthritis, thus addressing the issue of the added practical value of an informatics system. Finally, we describe how to integrate T-MedFusion with the Clinical Decision Support System (CDSS) in the hospital clinic.

### De-identification: Protecting patient privacy

The need for sharing and repurposing patient data in the translational research environment is constrained by several organizational and legal restrictions. One of the main barriers is the legitimate concern for protection of patient privacy [28]. It is therefore mandatory for any research institute to assure the protection of the patient information before making it accessible through any kind of platform. Coding processes approved by the local ethical committee were used for protection of identity of patients. Only a very limited group of named responsible clinicians/scientists have access to the coding keys, which allow identification of distinct individuals.

The de-identification process represents the first security layer in this system. This was complemented with additional layers to protect both privacy and the intellectual property rights and ownership of the database. In the next section we provide more details on the security protocol implemented in the system.

### Multilayer security approach

In the present test cases, the users of the system were granted the necessary credentials to access the coded data either inside the firewall of the Center for Molecular Medicine (CMM) at Karolinska University Hospital or as a remote user outside CMM. Adhering to good practice, the database server was isolated from the application server. This solution has both advantages and disadvantages and has been debated with regard to server consolidation and multitier architecture [29].

The data stored in T-MedFusion were completely de-identified: all individually identifiable attributes were removed and replaced with virtual attributes for joining different schemas and helping in multiple table queries. The system was installed on a Linux web server and accessible through a web browser. The user requires a credential provided by the Principal Investigator (PI) to log in to the system through the browser. All tasks executed by the user were tracked. Saving documents and operations like deleting and uploading data required additional privileges and permission as determined by the PI See figure S2.

Our system has therefore been designed to be PI driven, thereby ensuring control of data and use in the hands of the PI instead of the local or central IT staff. This came through as a very clear requirement in the interaction with the users as a paramount requirement in the design of the system. The PI control of the system, in conjunction with a user-friendly interface, were key factors for success, since the need for daily support from software engineering to use the system was alleviated. It is clear that without this PI driven design we would not have been able to proceed further.

### Improving cellular and immunological research in rheumatology

To assess the utility of the T-MedFusion platform for translational research, we asked whether the system could facilitate cellular and immunological research in the clinical rheumatology research unit. For example, it has hitherto been strenuous for researchers to systematically preselect cell samples based on clinical parameters.

At the rheumatology research laboratory, previous studies have been performed using donated patient material from Karolinska University Hospital. Patients donate several types of samples, primarily blood and synovial fluid, from which serum, DNA, and viable cells are retrieved and deposited in the RA biobank. The inclusion criteria for samples in a specific study, is typically based on serology and the availability of cells, while clinical information with regard to the samples is collected in the clinical setting and in the Swedish Rheumatology Quality of Care Registry; data from these sources are normally available to the cellular researcher only after specific search for information for each individual patients, something that has been a time-consuming and strenuous exercise.

Using our method, investigators are able to select research material based on the availability of the cell samples from the biobank, suitable genotype or serology status, and disease parameters from the clinical database (quality registry) in an efficient way. This facilitates and accelerates ongoing and future RA research at the clinical rheumatology research unit by enabling the identification of predictive markers such as immunological phenotypes.

For example, a common need is to be able to execute searches across several different data types using complex Boolean search criteria. As a rule, performing such queries requires each individual database to generate a list of hits. The next step is to combine these lists corresponding to the concatenated search criteria. This process is not only cumbersome but also prone to the introduction of errors. The simplified workflow supported by T-MedFusion, enabled by the introduction of methods instantiating data integration capabilities is as follows:

Pick a cell sample from the biobank based on the following criteria (Cx) using a Boolean search such that **C1** AND **C2** AND **C3** is true:

**C1**: Identify all samples where **HLA-DR** = ('*01/*04','*03/*04','*04','*04/*04','*04/*07','*04/*08','*04/*09','*04/*10','*04/*11','*04/*12','*04/*13','*04/*14','*04/*15','*04/*16').

**C2**: Only consider samples where the cell count is larger than 50×10∧6 cells.

**C3**: SNP rs2064476 = Nucleobase: Adenine & Guanine (AG) and Guanine & Guanine (GG) from genotype data source.

The result of the Boolean query is shown in table 2.

**Table 2:** The output from a Boolean query from three sources of data.

Rs2064476	Number of cells (10∧6)	Position	HLA-DR Type	cell source	CCP Serum
AG	13	01:F09	*04/*15	SFMC	438.9
AG	24	01:E10	*04/*15	SFMC	438.9
AG	17	01:H01	*04/*15	SFMC	238.9
AG	22	01:G02	*04/*15	SFMC	138.9
AG	53	01:B03	*04/*15	SFMC	538.9
AG	53	01:G04	*04/*15	SFMC	738.9
AG	31	01:H05	*04/*15	SFMC	738.9

Each row correspond to samples satisfying all three criteria (C1-C3). The position column pointed to the physical location of the cells in the freezer boxes. Cell source could be either synovial fluid mononuclear cells (SFMC) or peripheral blood mononuclear cells (PBMC), and the CCP-Serum column shows the serum levels of cyclic citrullinated peptide antibody.

Another example of workflow that also requires searches across different data sources is the challenge of determining how many patients are available with a certain human leukocyte antigen (HLA-DR) allele, antibody pattern, and disease severity from which cryopreserved cells, serum, and DNA samples are donated to the biobank.

A third example addresses the following question: Are there clinical differences (such as disease activity) between anti-CCP IgG versus anti-CCP single-positive and IgA/IgG double-positive RA patients? T-MedFusion provides the end users with a wide range of query capabilities to interact and retrieve the data from multiple data sources in a user-friendly and easy way. Tables 3-6 show example queries that are possible to perform.

**Table 3:** Retrieval of available peripheral blood mononuclear cell (PBMC) samples for each patient and the type of treatment the patient was undergoing at time of sampling.

Patient_ID								
Sample type	Number of cells	Treatment	Treatment Dose	DMARD1	DMARD1 Dose	Cortisone	Cortisone Dose	NSAID
PBMC	10	C	50 mg 7/d	Methotrexate	15 mg 0/			NSAID–COX1
PBMC	13,5	C	500 mg/d			Prednisolone	7.5 mg 1/d	
PBMC	10	C	500 mg/d			Prednisolone	5 mg 1/d	
SFMC	21,5	C	500 mg/d			Prednisolone	8.75 mg 1/d	
PBMC	13,5	C	50 mg 7/d	Methotrexate	15 mg 0/			

**Table 4:** Disease activity at different time points (visits to the clinic) for each patient in the cohort.

Patient_ID									
Pain scale	Patients' Global	Health assessment questionnaire	Swollen Joints	Tender joints	ESR	C-reactive protein CRP	Disease activity score DAS28	DAS 28 CRP	Doctor assessment
0	0	0	0	3	10	1	0	0	0
76	71	2	14	12	69	9,99	6,95	6,2	3
75	66	2	3	0	69	9,99	4,37	3,58	1
83	85	2,5	2	4	50	9,99	5,44	5,02	2
12	6	1,38	3	2	23	1	3,56	2,57	0

**Table 5:** Retrieval of cell samples according to HLA-DR type and sample date.

HLA, Dr04, cells per sample date				
Patient_ID				
RA Number	HLA Type	Sample date	Sample type	Number of cells
RA 1	*03/*08	Date A	SFMC	21,5
RA 2	*03/*08	Date B	PBMC	10
RA 3	*03/*08	Date C	PBMC	13,5

**Table 6:** Identification of the RA treatment type and dosage when the sample is collected from the patient.

Medication & sample							
Patient_ID							
Sample Type	Treatment	Treatment Dose	Bio Basmand 1	Cortisone	Cortisone Dose	DMARD1	DMARD1 Dose
Serum	C	50 mg 1/v	6	Prednisolone	2.5 mg 1/d	Methotrexate	20 mg 1/v
EDTA-plasma	C	50 mg 1/v	6	Prednisolone	2.5 mg 1/d	Methotrexate	20 mg 1/v
synovial	C	50 mg 1/v	6	Prednisolone	2.5 mg 1/d	Methotrexate	20 mg 1/v
DNA	C	50 mg 1/v	6	Prednisolone	2.5 mg 1/d	Methotrexate	20 mg 1/v
synovial	C	50 mg 1/v	6	Prednisolone	2.5 mg 1/d	Methotrexate	20 mg 1/v
DNA	C	50 mg 1/v	6	Prednisolone	2.5 mg 1/d	Methotrexate	20 mg 1/v

As demonstrated above, the methods introduced by the T-MedFusion system make it feasible to perform complex queries across distinct data types without requiring manual concatenation of lists obtained from elaborate searches in distinct databases. Significantly reducing the barriers to performing these kind of queries facilitates translational research in that in-house data can be reused and investigated much more thoroughly.

### Extract variation in antibody patterns

**Figure 5:**
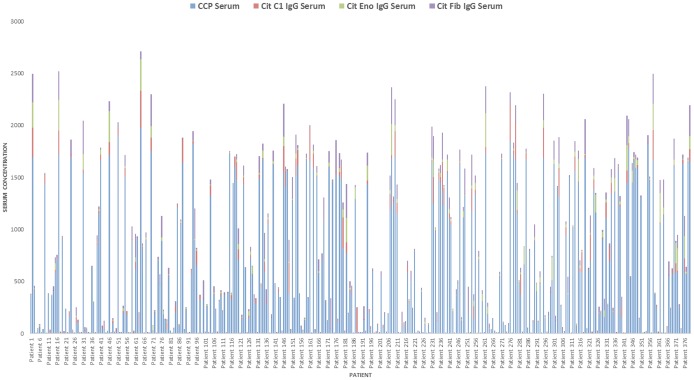
RA patient's profile for anti-citrullinated protein antibodies (ACPA). Each column in the x-axis represents one patient with four different serum concentrations. The y-axis represents concentration levels of the different serums (CCP, Cit C1 IgG, Cit Eno IgG and Cit Fib IgG).

In addition to performing complex queries across distinct databases, investigators often want to extract time series patterns for longitudinal cohorts from either one or several data sources. Our system facilitates such analysis. For example, to explore the level of anti-citrullinated protein antibodies (ACPA) for an entire cohort and establish longitudinal cohort variation in antibody patterns, we can visually inspect the data in figure 5. For established longitudinal cohorts in RA or any other disease, the extraction and visualization of the time series will enable the investigator to study which fine specificities the anti-citrulline response displays (i.e., beyond the CCP-test). This method could be applied to any similar data for a cohort of patients.

### Studying biomarker predictors of treatment response in RA patients

Recently, several effective immunologically designed treatments, commonly referred to as biological, have become available in the standard care of RA patients, improving their prognosis profoundly. However, the disease course varies widely among patients, and it is also clear that each of the new biological therapies is usually very effective in only a fraction of patients [30]. Non-responders to one specific therapy may, however, still respond to another drug, in part because it modifies another pathogenic pathway, which then may presumably be more prominent in the disease pathogenesis in that particular patient. Therefore, there is an urgent need in the clinic to identify (molecular) biomarkers that can predict the response to specific treatments at baseline. This may therefore support the accurate decision in selecting the correct treatment from the start, and the changes to biomarkers over time may also yield valuable information about the effects of such treatments. The T-MedFusion system is poised to be useful in the identification of such biomarkers, since the investigator can cross-link a response to therapy from the register-based follow-up data with molecular information collected in the research unit.

For example, with the availability of large numbers of biological samples collected at different time points for patients, an important issue for clinicians and investigators is to select samples according to predefined criteria within a defined time window for follow-up, in order to match those to clinical response data. One example is the selection of serum samples for RA patients at the baseline visit at treatment start and also at a follow-up visit, having available data on the outcome measure, e.g., disease activity scores (DAS) at these time points. To answer such a query, users need to extract the following information: treatment and DAS-related parameters from the clinical database, as well as sample and temporal information (sample donation date and time) from the biobank. The following algorithm describes the scenario of seeking serum samples in order to study the predictive value of biomarkers on patient response to treatment from the quality-of-care registry. From the RA biobank we have the following data:

- Total number of patients recruited in the study = 1622 active patients in the clinical database.
- Total number of serum samples in the biobank = 30,765. The samples are collected at the RA clinic and stored in the RA biobank at the CMM.

1. Identify and retrieve the clinical data for the cohort.
2. Extract treatment and DAS data for the selected cohort.
3. Identify and retrieve serum samples for the cohort in step 1.
4. Fuse the results from steps 1 and 3.
5. The result from step 4 is 22,279 (serum samples) out of 30,765 samples (according to predefined criteria on the serum sample volume and status in the freezer).
6. Extract all patients under treatment A = 1276 patients. Then extract two types of serum for this cohort according to:
  - Initiation of DAS time window: Sample is selected if the sample has been collected at most 30 days before the DAS start date or up to 40 days after: 3204 serum samples satisfied this condition.
  - End DAS time window: Sample is selected if the sample has been collected at most 75 days before the DAS end date or up to 152 days after: 1952 serum samples satisfied the condition.
7. Repeat step 6 for treatment B, 95 patients: found 30 samples at Start DAS time window and 24 at End DAS time window.
8. Repeat step 6 for treatment C, 251 patients: found 215 samples at Start DAS time window and 221 at End DAS time window.

The system provides a summary of the above findings, as illustrated in table 7.

**Table 7:** Summary of available serum samples in the RA biobank.

Treatment groups	Total number of patients	All serum samples	StartDAS		EndDAS	
			samples	patients	samples	patients
A	1276	22,279	3204	313	1952	288
B	95	22,279	30	9	24	5
C	251	22,279	215	53	221	36

Total number of patients and the classification of the samples according to the DAS time window (start and end).

Without the integration capabilities of the T-MedFusion system, the above workflow would have been difficult and time-consuming to execute. Our methods readily thus enabled an integrative analysis of biomarkers corresponding to the following therapeutics: Infliximab, Adalimumab, Etanercept, Methotrexate (MTX).

### Cohort stratification using waterfall filtering

**Figure 6:**
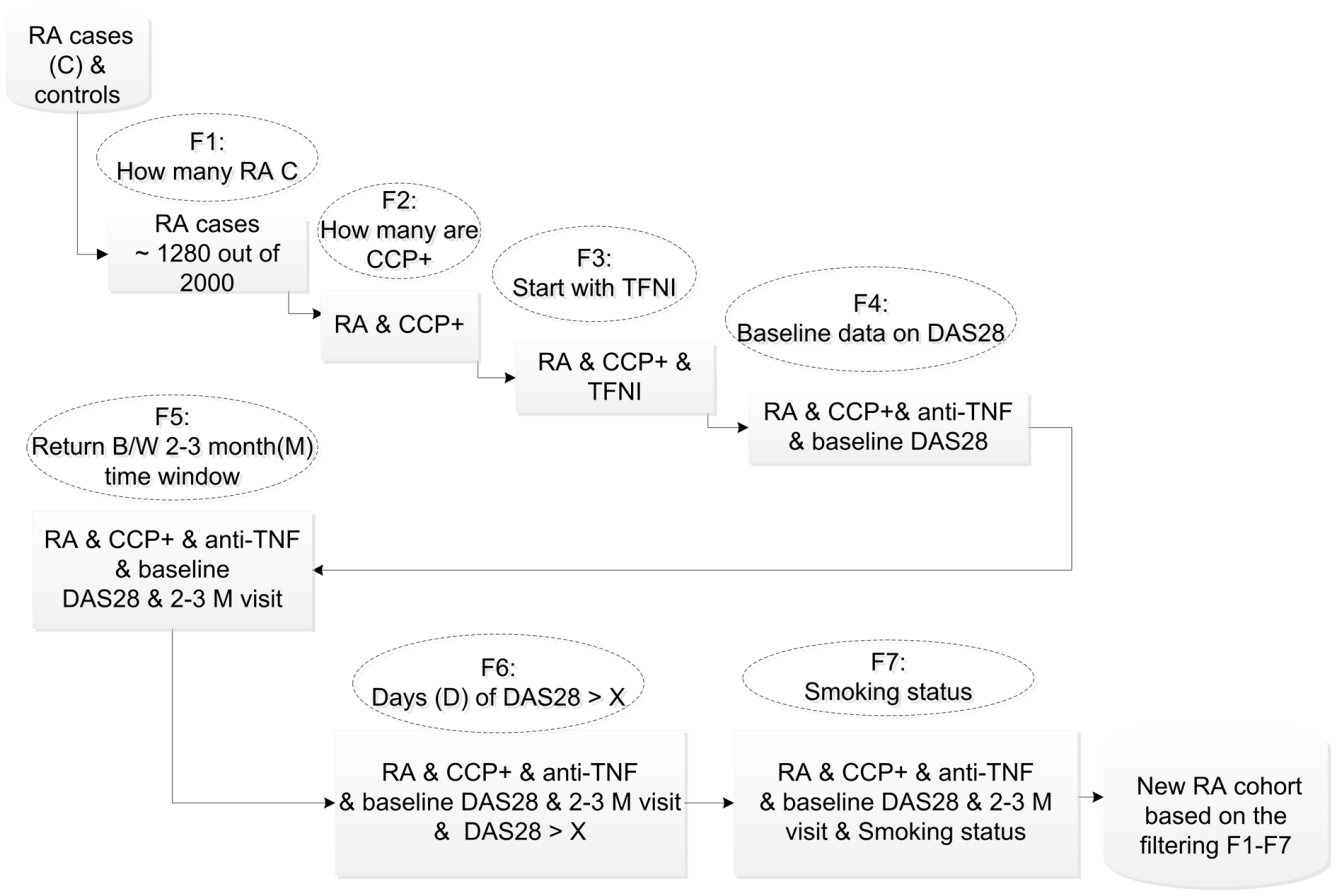
A waterfall query model to filter and stratify the patients according to specific criteria.

In many cases an investigator needs to run a multi-criteria filtering process and stratify the research cohort. This type of filtering requires a complex query method. Through a waterfall filtering process, T-MedFusion supports patient stratification, as illustrated in figure 6. In this case the user wants to find all RA cases who are anti-CCP+ (having antibodies against cyclic citrullinated peptides), start with anti-TNF (anti-tumor necrosis factor) treatment, and have a baseline DAS28 with a follow-up visit in the two- to three-month time window. The system calculates the change in DAS28 and further filters the patients to a specific DAS28 value (X).

### Exploration of metabolic profiles for psoriasis patients

Next we sought to evaluate the applicability of T-MedFusion methods in another disease area, namely translational research on psoriasis. We therefore created a database structure capable of storing the metabolic profiles of psoriasis cases and controls, with the aim being to explore whether a metabolic profile in a subset of psoriasis patients is associated with SNPs identified in other studies, such as SNPs of relevance for cardiometabolic disease and diabetes. Criteria that need to be fulfilled in the first round are the following:

Criteria of obesity and hyperlipidemia: BMI>29; LDL>4.3; Triglycerides>2.6, the total number of patients = 747 patients. The result is illustrated in table 8.

**Table 8:** Metabolic profile of psoriasis patients.

Question/condition	Number of patients	Criteria
BMI>29	92	BMI
BMI>29 & LDL>4.3	6	BMI & LDL
BMI>29 & LDL>4.3 and Triglycerides>2.6	0	BMI & LDL & Triglycerides
LDL>4.3	85	LDL
LDL>4.3 & Triglycerides>2.6	4	LDL & Triglycerides
Triglycerides>2.6	36	Triglycerides

Note: The criteria were BMI>29; LDL>4.3; Triglycerides>2.6 hyperlipidemia.

In the second round the criteria to be fulfilled are: BMI<25, Triglycerides<2 and LDL<4.

Total number of patients = 747 patients.

The results are illustrated in table 9.

**Table 9:** Metabolic profile of psoriasis patients.

Question/condition	Number of patients	Criteria
BMI<25	379	BMI
BMI<25 & Triglycerides<2	359	BMI & Triglycerides
BMI<25 & LDL<4	335	BMI & LDL
BMI<25 & Triglycerides<2 & LDL<4	321	BMI & Triglycerides & LDL
Triglycerides<2	676	Triglycerides
Triglycerides<2 & LDL<4	556	Triglycerides & LDL
LDL<4	611	LDL

Note: The criteria were BMI<25, Triglycerides<2 and LDL<4.

For the entire cohort the system allows the researcher to visually explore the change in the psoriasis area severity index (PASI) at time 00 and after 10 years. PASI is a quantitative measurement of the severity of the skin disease, integrating involvement, redness, and thickness of lesions. In conclusion, our method permits a longitudinal analysis of a patient's metabolic profile.

### Integration with clinical decision support system

At the RA clinic a clinical decision support system (CDSS) generated within the Swedish Rheumatology Quality of Care Register supports the doctors in their decisions about diagnosis, status of disease, and medication plan. The CDSS provides a simplified view of the patient's illness, treatment, and outcome. Currently the CDSS is based on data from the Quality of Care Registry, which also includes selected clinical laboratory test results. However, there is a need to integrate the clinical data and lab data with additional molecular data and findings from research.

Therefore, the current architecture of T-MedFusion was designed to provide a high level of interoperability with different bioinformatics tools and workflows. This is possible through the so-called service-oriented architecture (SOA) [31]. The main feature of the SOA is enabling the reuse of information while maintaining a soft connection between the end users of the information platform (service consumer) versus the generators (systems). Using this architecture model the system can be interoperable with other infrastructures, thus making it possible to exchange different types of data stored in T-MedFusion with data stored in other systems. To illustrate this point we have performed a system-level integration between T-MedFusion and the RA CDSS (from the Quality of Care Registry) at Karolinska University Hospital.

The integration of T-MedFusion with the CDSS for RA at this stage will propagate the results of the ACPA tests from the research lab to the clinic. The knowledge about whether the patient is ACPA positive or negative is an important predictor for the need for result of treatment with some biologicals used in RA [32]. T-MedFusion can therefore enable the translation of the research results into clinical practice for predicting the eligibility of the patient for a specific treatment.

To introduce such information into the health-care system, the T-MedFusion transfers the research results about ACPA directly into the CDSS. The level of anti-citrullinated protein antibodies (ACPA) includes four parameters:

- CCP serum: Cyclic citrullinated peptide (CCP) - a diagnostic test for RA.
- Cit C1 IgG Serum: Cit C1 immunoglobulin G serum.
- Cit Eno IgG Serum: Cit enolase immunoglobulin G serum.
- Cit Fib IgG Serum: Cit fibrinogen immunoglobulin G serum.

The variant of RA (the ACPA-positive RA subset), is one of the genetically best characterized disease variants that exist, and a large number of studies have confirmed the effects of the genetic variants in RA diagnosis and treatment. Our entire approach is to firstly allow the clinicians to use the novel analyses of genetically complex diseases where different molecular phenotypes, here fine specificities of autoantibody re-activities, can be investigated. A second goal is to create a bridge between bench and bedside and translate research results to clinical care. Research wise, there has been very rapid development in this field recently (see [33], [34], [35]). All this combines to make the case of RA a very good example to demonstrate here.

**Figure 7:**
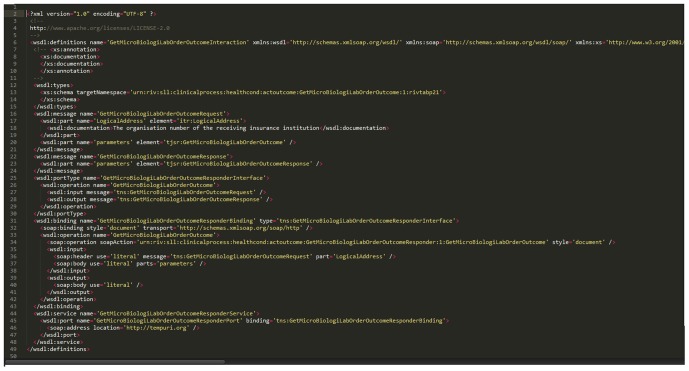
Segment of the WSDL file used to describe the SOAP service for integration T-MedFusion with CDSS.

**Figure 8:**
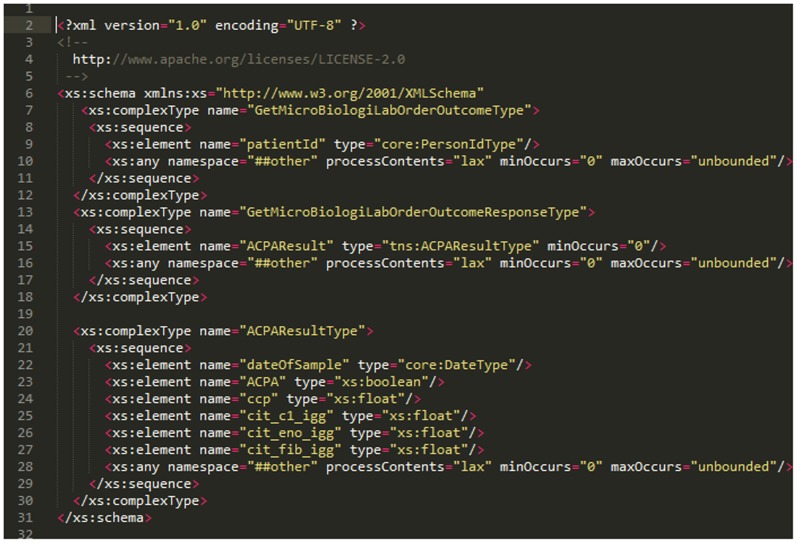
The XML XSD file defining the basic element send from T-MedFusion to the CDSS via the SOAP services defined in the WSDL file.

**Figure 9:**
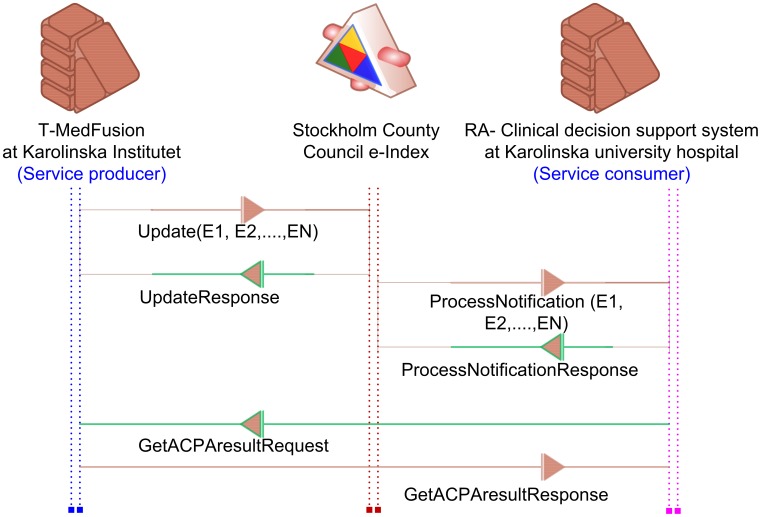
T-MedFusion (service provider) and the RA CDSS communicating through the Stockholm County Council exchange index (e-index). The e-index monitors the update service from T-MedFusion. Whenever a new ACPA result arrives, the e-index sends a notification to the CDSS. The CDSS sends a GetACPAresultRequest to T-MedFusion and as response obtains ACPA results for one or several patients. Streaming of the response and request services and communications between the systems are conducted through exchange of security certificates.

The integration of T-MedFusion with the CDSS was achieved through the service integration platform hosted by Stockholm County Council as part of a specific project named 4D, where RA serves as a pilot project. The integration, using a web service based on requests and responses from the XML-based messaging protocol SOAP, was defined and agreed on in a service contract. The service contract (SC) is a technical agreement between the service provider (T-MedFusion) and consumer (CDSS). The exchange format and service communicate through service-oriented architecture and XML schemas. The SC consists of a web service description language (WSDL; figure 7) file with the associated XML schemas (XSD; figure 8), which is a document that describes all the rules, syntax and elements provided in the contract. The workflow and communication between the T-MedFusion and the CDSS is illustrated in figure 9.

### A data-sharing model

T-MedFusion addresses researchers' challenges to maintain and manage research data in a long-term and persistent manner [36]. The system enables simplified procedures for uploading data from different formats. In addition to the database management, the implementation of T-MedFusion increases collaboration possibilities and the sharing of research data and results across different research groups working in similar or different therapeutic areas. It also facilitates collaboration between biologists and bioinformaticians to further run advanced bioinformatics and computational modeling analysis. This since the process of accessing and mapping which data is available is significantly simplified, as this step is as a rule a major hurdle in numerous inter-disciplinary collaborative projects.

### Outcome of the technical performance study

The results of the performance test are illustrated in data file (**SM: Data file S1. Performance evaluation results**) and table 10. We recorded the average response time in seconds for active users, http errors, and received and sent kbit per second. The maximum response time was found to be 0.12 second; no errors were detected. We observed some peaks during the second day of the test because heavy computation was run on the server, which took a lot of CPU time and memory.

**Table 10:** Result of performance evaluation: Test execution parameters:Test status: finished, Test started at: 6/7/2012 8:04:55 PM, Test finished at: 6/12/2012 8:04:55 PM, Test duration: 120:00:00 hours (five days), Virtual users: 1–20.

System element	Total number of users	User think time in seconds	Response time, sec (with page elements)		HTTP errors (%)	Network errors (%)	Timeouts (%)
Response time in seconds for the user to load the login page	20	0	Minimum	Maximum	0	0,000034	0
			0.01	5.09	0	0,000034	0
Response time for the system user to log in to the system	20	0–8	0.03	5.13	0	0,000034	0
Response time to load the cell (from the biobank) and medication (from EMR)	20	0–5	0.76	38.3	0	0,000034	
Response time to search for patient's cell (from the biobank)	20	0–7	0.01	5.07	0	0,000034	0
Response time to get suggestions for search parameters from the biobank	20	0–9	0.11	27.6	0	0,000034	0
Response time to get suggestions for search parameters from the EMR	20	0–0	0.11	24.9	0	0,000034	0
Response time to get suggestions for search parameters and select one or more parameters	20	0–0	0.11	5.27	0	0,000034	0
Response time to get the final results of the query.	20	0–5	0.33	33.8	0	0,000034	0

The results show that the system response time is of sufficient quality and scales up with increasing workload (number of virtual users from single user up to twenty users at the same time).

## Discussion

Our work as illustrated in this case study, effectively integrates requirements from the clinical and the software engineering perspectives in a translational medicine setting. The three principal achievements can be summarized as (a) a design of a user-friendly web-based investigator-driven system for data management and integration, while adhering to proper de-identification and security measures, using a modular three-layer architecture; (b) providing a real-world test of the system using clinical cohorts of rheumatoid arthritis and psoriasis; and (c) demonstrating interoperability, in that the system supports and integrates with a clinical decision support system for health care.

Below we elaborate on the challenges and opportunities in pursuing clinically driven informatics development, which includes developing appropriate tools facilitating data quality control and curation, data import, data integration, and possibilities for generalization to other disease areas while still having a modular and interoperable system. We close the discussion by some final remarks on future developments.

A clinically driven approach to integrate clinical and biomedical databases entails a number of challenges that might not have been readily apparent if a purely software engineering approach had been at work. For example, to have a system where the PI is in control of the environment is of uttermost importance, which should be considered together with the need for a user-friendly system enabling daily use by non-computer experts. To identify and meet those needs we therefore developed the methods and algorithms in close dialogue with clinical researchers in clinical settings. For example, we do not require the users to write query statements in scripting languages and thereby spend time dealing with syntax and computer commands. We augmented the query interface with information visualization methods to support cohort discovery through data visualization techniques. Moreover, we introduced a waterfall model to query and filter the cohorts using a wide range of inclusion and exclusion criteria. Finally, T-MedFusion reduces the required time to preselect biological samples based on clinical parameters such as disease activity and medication. The user interface allows the preparation of the research material according to the availability of the samples, suitable genotype or serology status, and various disease parameters.

Yet this work raised several challenges. It became clear that there is an urgent need for development of semi-automatic tools to facilitate the tasks such as quality control of data, import of data, identification of inconsistencies, and annotation. Such tools should be user-friendly in the hands of the clinical and biomedical scientist, while still being grounded in solid software engineering. Current systems targeting monitoring of clinical trials, [8] and [11], currently require sophistication in computer science beyond that of the translational scientist. Hence, extracting, importing, and loading data through the graphical user interface of the T-MedFusion system has been simplified by providing a wizard-based interface for importing data sets generated in the research lab (in several formats) into the system, thus enabling the biomedical scientist to perform data import without dealing with a command shell tool like Oracle SQL*Loader (sqlldr) [37], or an ETL tool like Pentaho (Kettle) data integration software package, in which the users need proper training in the design of metadata and taxonomy [38].

For a more extensive discussion of remaining challenges with respect to medical informatics systems, see [13] and [12]. An additional obstacle is that the quality and reusability of the data is also dependent upon proper annotation, which currently requires hands-on knowledge of the particular biomedical domain, generally beyond the abilities of the software architect. Hence, it is a challenge to develop methods that can mitigate this gap between the importance of annotation and the current more or less manual procedures for performing this work.

To support the practical demands from the users, we benefited from requirement engineering methods to determine their requirements. This guided our development and modification of techniques for data integration and system architecture. This is also important since we aim to design a system amenable to extension into other disease areas. Integration of several databases for large cohorts creates opportunities to query and understand relationships among the different data sets collected for the same patient. To achieve this feature, our methods provide a wide range of query capabilities with a usable interface. The main challenge met by the T-MedFusion system is the need for an underlying system architecture in which information can be stored, queried, accessed, annotated, and shared in numerous ways, while still respecting that different end users have different needs in terms of interface and research workflow.

We anticipate that the kind of system we have developed can readily be extended to cover other translational areas of research within the Karolinska University Hospital in a first wave of development. At a later stage T-MedFusion may serve as a mature platform that could be connected with transnational efforts at an international level. As a proof of principle we have presented the integration to the health care system and tools for clinical decision support, thus suggesting that the T-MedFusion platform can communicate readily with other national and transnational systems. This user-friendly design geared towards translational research has to a large degree approximated the components and functionalities of T-MedFusion, thereby serving as a “local” integrative and interoperable informatics platform.

There are several potential applications for the methods we introduced beyond the ones we have illustrated using RA and psoriasis. For example, in the cardiovascular disease area we have implemented the methods to study biomarkers for venous thromboembolism (VTE). In that project, clinical investigators are for example collecting lifestyle information for about 200 patients to be integrated with their EMRs, laboratory and screening tests, and VTE treatment. We are currently testing the same methods to integrate clinical data targeting approximately 150 patients diagnosed with lung cancer (non-small-cell lung carcinoma). Here our aim is to predict the outcome of stereotactic body radiation therapy (SBRT) [39], including induced side effects and toxicity. Furthermore, we are currently assessing whether it is feasible to apply the same architecture model to manage the breeding and associated genetics databases associated with experimental animal models.

Translation informatics systems should provide a technology-independent model of integration, which we illustrated in the last part of the Results section by describing how the service contract, defined as a set of rules governing the integration requirements between service consumers and service providers, facilitates interaction between researchers and clinicians and permits a secure flow of data between the bench and the clinic for an RA patient.

By providing interoperable and scalable systems as T-MedFusion, we aim to support researchers and clinicians in better understanding the capability of information system and software engineering to translate basic research results into clinical practice. Without appropriate understanding of the end users' needs at the clinic and in biomedical research, it is difficult to achieve progress. Likewise, nontechnical issues of ethics and security are highly important for gaining trust, facilitating the flow of information from bench to bedside, and providing the quantitative and high-quality data on patients and diseases that translation medicine critically depends on. Some sort of software infrastructure, such as T-MedFusion, is a prerequisite for repurposing clinical data into the research setting. In our continued work, we intend to improve the tool support for enabling and managing genomics and proteomics data from high throughput platforms. We expect that the fusion of different types of clinical information with rich omics data will improve the understanding of complex diseases and provide new insights into treatment methods and the efficacy and toxicity of drugs. Translational medicine is becoming increasingly dependent upon data captured in health care, clinical research, and molecular research. Yet there is an urgent need for integrative, interoperable informatics systems mitigating current barriers, thereby enabling systems analysis of diseases and therapeutic interventions. We conclude that the system can readily be used in a translational medicine setting in the hands of clinicians or biomedical researchers, while still being built according to robust and interoperable software engineering principles.

## Supporting Information

Figure S1 User interface for SNP data stored in MedFusion.
(TIF)

Figure S2 Schematic description of the security layers of T-MedFusion.
(TIF)

File S1 Performance evaluation results.
(XLSX)
