# Supplementary figures and images for: Accelerating Translational Research by Clinically Driven Development of an Informatics Platform–A Case Study
Source: PLoS One. 2014 Sep 9;9(9):e104382. doi: 10.1371/journal.pone.0104382 (PMC4159182; doi:10.1371/journal.pone.0104382)

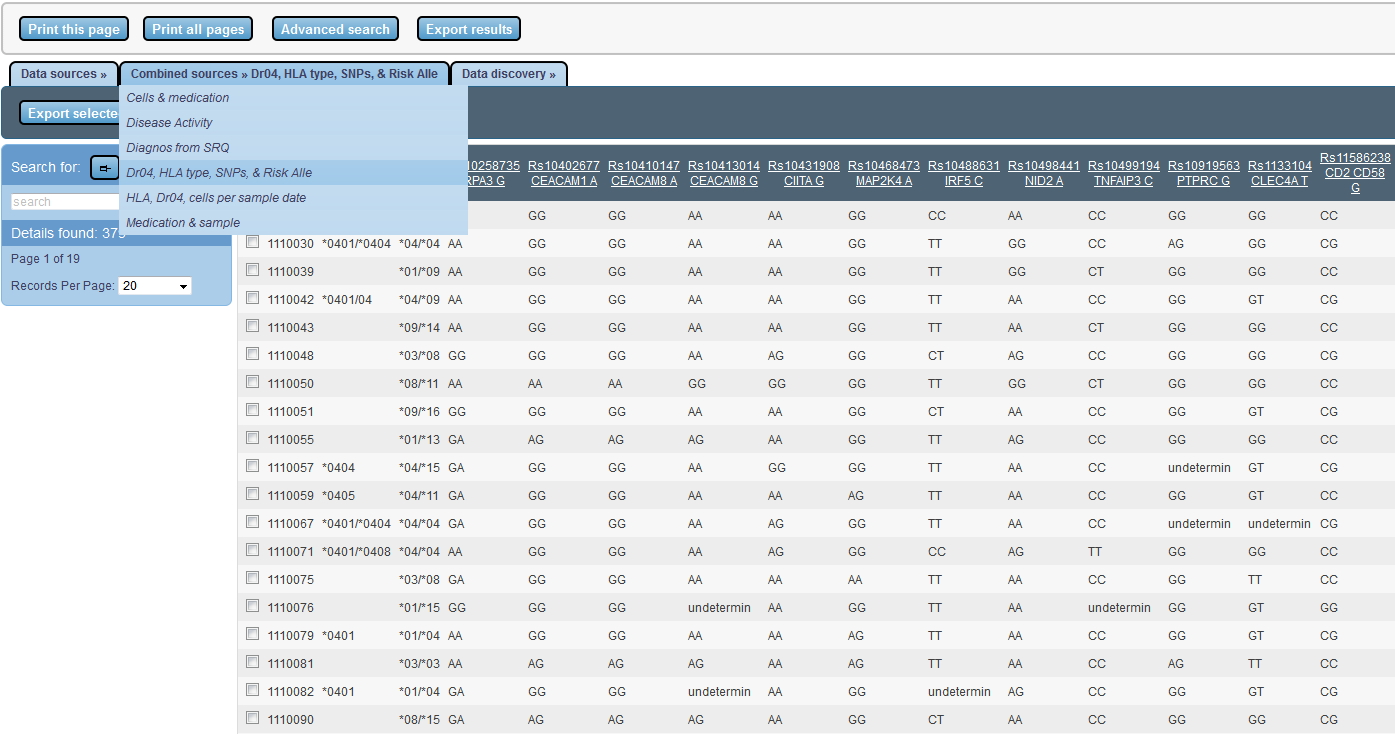

Supplement: Figure S1 — User interface for SNP data stored in MedFusion. (TIF) [file pone.0104382.s001.tif]

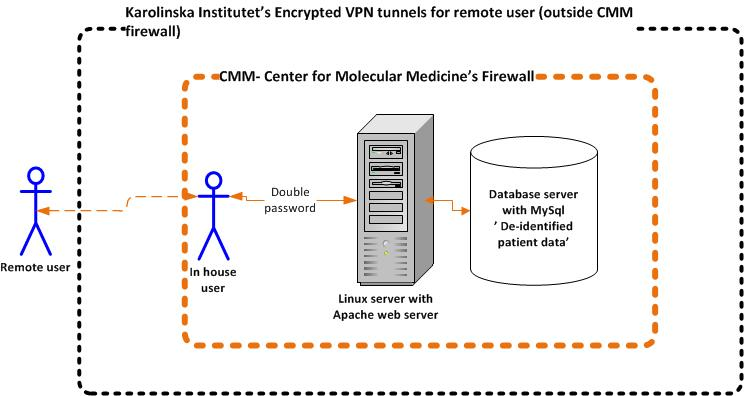

Supplement: Figure S2 — Schematic description of the security layers of T-MedFusion. (TIF) [file pone.0104382.s002.tif]
